# Spectroscopic, electrochemical, thermodynamic and theoretical insights into solvent effects for the intensification of the modified OxFA process

**DOI:** 10.1039/d6ra02899c

**Published:** 2026-04-30

**Authors:** Jan-Dominik H. Krueger, Pegah Saedi, Maximilian J. Poller, Dzmitry H. Zaitsau, Riko Siewert, David Robinson, Karsten Müller, Jakob Albert

**Affiliations:** a Institute of Technical and Macromolecular Chemistry, Universität Hamburg Bundesstrasse 45 20146 Hamburg Germany jakob.albert@uni-hamburg.de; b Lehrstuhl für Technische Thermodynamik, Universität Rostock Albert-Einstein-Str. 2, Haus 1 18059 Rostock Germany; c Department of Chemistry and Forensics, School of Science and Technology, Nottingham Trent University Clifton Lane Nottingham NG11 8NS UK

## Abstract

Producing short-chain carboxylic acids like formic acid (FA) from biomass in the OxFA process is a promising strategy for a green and sustainable chemical industry. Herein, we employed spectroscopic (NMR, UV-vis, and GC-MS), electrochemical (CV and SWV), and thermodynamic (gas solubility) measurements in combination with theoretical modelling by DFT for rationalizing the dominating effects of co-solvents on the catalytic efficiency of xylose oxidation to FA in the modified OxFA process catalyzed by H_8_PV_5_Mo_7_O_40_ (HPA-5) polyoxometalate. Specifically, the effect of oxygen solubility in different solvent mixtures in combination with the redox potential of the first reduction event of HPA-5 during the electrochemical treatment revealed the combined effect of both thermodynamic and catalytic properties on the effective reaction kinetics for xylose oxidation to FA. Therefore, the ease of xylose oxidation is directly linked to the redox potential observed in different co-solvent mixtures. Based on these insights, systematic optimization of the reaction parameters in the most promising water-acetonitrile solvent mixture using design of experiments (DoE) and a central composite design (CCD) achieved an FA yield of 90% at full xylose conversion, with only 5% CO_2_ formation after 2 hours of reaction. The insights from this study provide a strong foundation for future process intensification in biomass valorisation technologies.

## Abstract 2

The effects of co-solvents on the catalytic efficiency of xylose oxidation to formic acid in the modified OxFA process were explored by a combination of spectroscopic, electrochemical and thermodynamic measurements, as well as DFT modelling.

## Graphical abstract

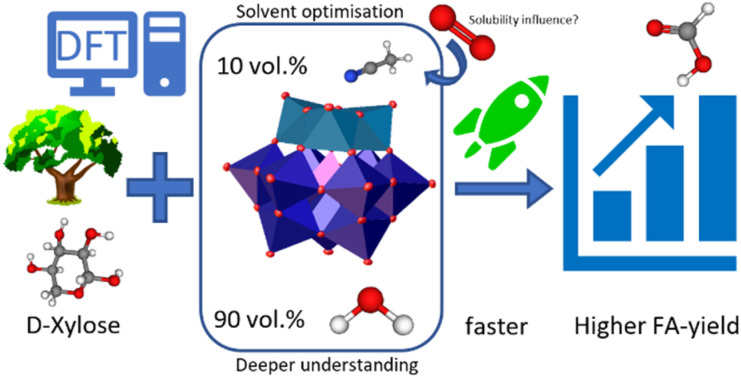

## 1 Introduction

The increasing depletion of fossil resources coupled with growing environmental concerns has intensified the search for renewable alternatives in chemical production. Biomass, being abundant, renewable, and currently underutilized, presents a promising feedstock for sustainable chemical synthesis, offering a path towards reduced reliance on finite resources.^1,2^ Its use aligns with global efforts to foster environmental protection and resource conservation, making the conversion of biomass into platform chemicals a vital strategy in advancing the circular bioeconomy.^3,4^

Platform chemicals serve as versatile intermediates that can be transformed into a wide array of value-added products, representing a cornerstone of sustainable chemical industries.^5^ Examples such as 5-hydroxymethylfurfural, levulinic acid, and furfural exemplify the potential of biomass-derived compounds to replace petrochemical counterparts.^6,7^ The development of efficient conversion technologies, including biochemical,^8,9^ thermochemical,^10,11^ and catalytic processes,^12,13^ though promising, faces challenges such as low product yields, catalyst deactivation, and high production costs.^14,15^ Overcoming these hurdles through advances in catalysis and process integration is crucial for industrial-scale implementation, which in turn can significantly impact both our economy and society by promoting green chemistry and reducing environmental footprints.^16,17^

The sustainable transformation of lignocellulosic biomass into value-added chemicals is a central challenge in green chemistry, where C_5_ sugars, primarily xylose and arabinose, are crucial feedstocks in biomass conversion processes because they constitute a significant fraction, between 20% and 35%, of lignocellulosic biomass, especially within the hemicellulose segment.^18,19^ However, technical challenges, such as microbial preferences and process bottlenecks, limit their efficient utilization, requiring innovative processes.^20,21^ The inherent recalcitrance and heterogeneity of hemicellulosic biomass often limit the conversion efficiency and product selectivity.^22^

Modern advances in catalytic and oxidation technologies have enabled high-yield, selective processes from lignocellulosic and carbohydrate-rich feedstocks, facilitating efficient and scalable production of platform chemicals.^23–26^ Especially, the OxFA process converts biomass efficiently into the chemical hydrogen storage compound formic acid (FA) by using polyoxometalates (POMs) with the general formula H_3+*n*_PV_*n*_Mo_12−*n*_O_40_ (*n* = 2–5) as homogeneous catalysts and molecular oxygen as the oxidant in aqueous solutions.^27,28^ FA is emerging as a promising product due to its remarkable versatility, sustainability, and critical role in advancing green energy applications.^29,30^ Its widespread industrial uses span agriculture, textiles, food preservation, and leather processing, and it serves as a key chemical intermediate, demonstrating substantial market demand,^30,31^ and can work as a safe, easily handled liquid hydrogen carrier.^32,33^

Recent studies have demonstrated that the use of organic co-solvents – such as alcohols (methanol, ethanol,^34,35^ and isopropanol^36^) and dimethyl sulfoxide (DMSO)^37^ – as additives in aqueous solutions can have a significant impact on the efficiency of FA formation from the biomass. These organic co-solvents not only enhance substrate solubilization and catalyst accessibility but can also modulate the redox behaviour and stability of POMs, leading to enhanced reaction kinetics and improved selectivity to FA.^38–40^ It has been suggested that alcohols such as methanol and isopropanol can act as radical scavengers, suppressing overoxidation and thus increasing the yield and selectivity of formic acid.^41^ Recently, it has been discovered that methanol can inhibit the over-oxidation of intermediates by forming hydrogen bonds with the active vanadium centres in the POM moiety, competing with xylose binding.^42,43^ Additionally, acidic additives, including oxalic acid and acetic acid, were found to accelerate substrate conversion, but can also compete with the active vanadium centres in HPA-2 (H_5_PV_2_Mo_10_O_40_), thereby inhibiting the catalytic activity.^44^ As a result, the integration of the most efficient HPA-5 (H_8_PV_5_Mo_7_O_40_) POM catalyst with the tailored solvent and additive systems represents a promising strategy for the efficient and sustainable production of FA from renewable biomass resources.^45,46^

This study therefore aims at identifying the most promising organic co-solvent and at optimising the reaction parameters for its application in the selective catalytic oxidation of xylose to FA. This encompasses testing the performance of different solvent mixtures and determining kinetic parameters for the POM-catalyzed oxidation of xylose to FA. The catalytic results will be correlated with the oxygen solubility measurements of the different solvent mixtures and electrochemical potential measurements of the HPA-5 catalyst. For a more in-depth mechanistic understanding, DFT calculations were performed. Finally, the reaction conditions for the most effective solvent mixture were optimised using the systematic experimental approach of a Design-of-Experiments (DoE) study.

## 2 Results and discussion

Building on previous studies discussing the influence of several co-solvents on the catalytic performance of the POM-catalyzed biomass oxidation to FA in aqueous solutions,^33–44^ a systematic study using xylose as a model substrate was carried out to verify the different effects that the co-solvents exert on the HPA-5 catalyst. Specifically, a series of polar protic co-solvents like MeOH, EtOH, *n*PrOH, *i*PrOH, *n*BuOH, *s*BuOH, *t*BuOH and ethylene glycol as well as polar aprotic co-solvents like DMSO and MeCN, and the ketones acetone and 2-butanone were added in an amount of 10 vol% to an aqueous solution.

### 2.1 Selection of suitable co-solvents

First, the impact of various co-solvents on the stability and catalytic performance of the HPA-5-catalyzed xylose oxidation reaction under 50 bar O_2_ and 100 °C was investigated, paying particular attention to the stability of the co-solvent under reaction conditions. Xylose conversion and product yields for all tested co-solvents after 5 h reaction time are compared in Table 1, whereby product yields are referenced to the initial xylose concentration used in each experiment (yields > 100% can be led back to decomposition of the used co-solvents).

**Table 1:** Selection of suitable co-solvents for the modified OxFA process. Reaction conditions: *c*(xylose) = 25 mmol L^−1^, *c*(HPA-5) = 15 mmol L^−1^, *V* = 45 mL, *p*O_2_ = 50 bar, *T* = 100 °C, *V*_stirrer_ = 1000 rpm, *t* = 5 h, and solvent composition: 100% water or 90% water + 10% co-solvent by volume = MeOH, EtOH, *n*PrOH, *i*PrOH, *n*BuOH, *s*BuOH, *t*BuOH, 2-butanone, acetone, DMSO and ethylene glycol. All yields referenced to xylose conversion

Additive	*X*_xylose_ (%)	*Y*_FA_ (%)	*Y*_CO2_ (%)	*Y*_CO_ (%)	*Y*_AA_ (%)
—	100	63	18	—	—
MeOH	100	97	2	—	—
EtOH	100	118	5	—	6
*n*PrOH	100	170	10	6	20
*i*PrOH	100	111	2	—	2
*n*BuOH	100	248	24	17	28
*s*BuOH	100	265	88	2	260
*t*BuOH	100	102	5	—	6
Ethylene glycol	100	330	14	—	—
Acetone	100	134	10	—	18
DMSO	100	130	2	2	—
2-Butanone	96	213	119	—	628
MeCN	96	85	9	—	6

All experiments conducted with a reaction time of 5 hours resulted in the complete conversion of xylose. An interesting observation was the formation of the by-product: acetic acid (AA). This was not observed in xylose oxidation in previous studies^21,44^ and can therefore serve as an indicator of co-solvent instability. Out of the tested solvents, *n*PrOH, *n*BuOH and acetone showed acetic acid yields (*Y*_AA_) ranging from 18% to 28%, indicating that these co-solvents are somewhat unstable under the applied reaction conditions. In contrast, *s*BuOH and 2-butanone demonstrated extraordinarily high *Y*_AA_ values of 260% and 628%, respectively (normalised to the xylose concentration used), indicating significant degradation of the co-solvent and instability within the reaction environment. Elevated FA yields (*Y*_FA_ > 100%) and CO_2_ formation point to concurrent oxidative and C–C cleavage reactions, whereby the co-solvent itself appears to act as an additional substrate. This phenomenon was observable for acetone, DMSO, and most notably, ethylene glycol. However, DMSO exhibits only minimal CO_2_ formation and is suitable for studies aimed at enhancing selectivity and reaction kinetics.^37^

**Fig. 1:**
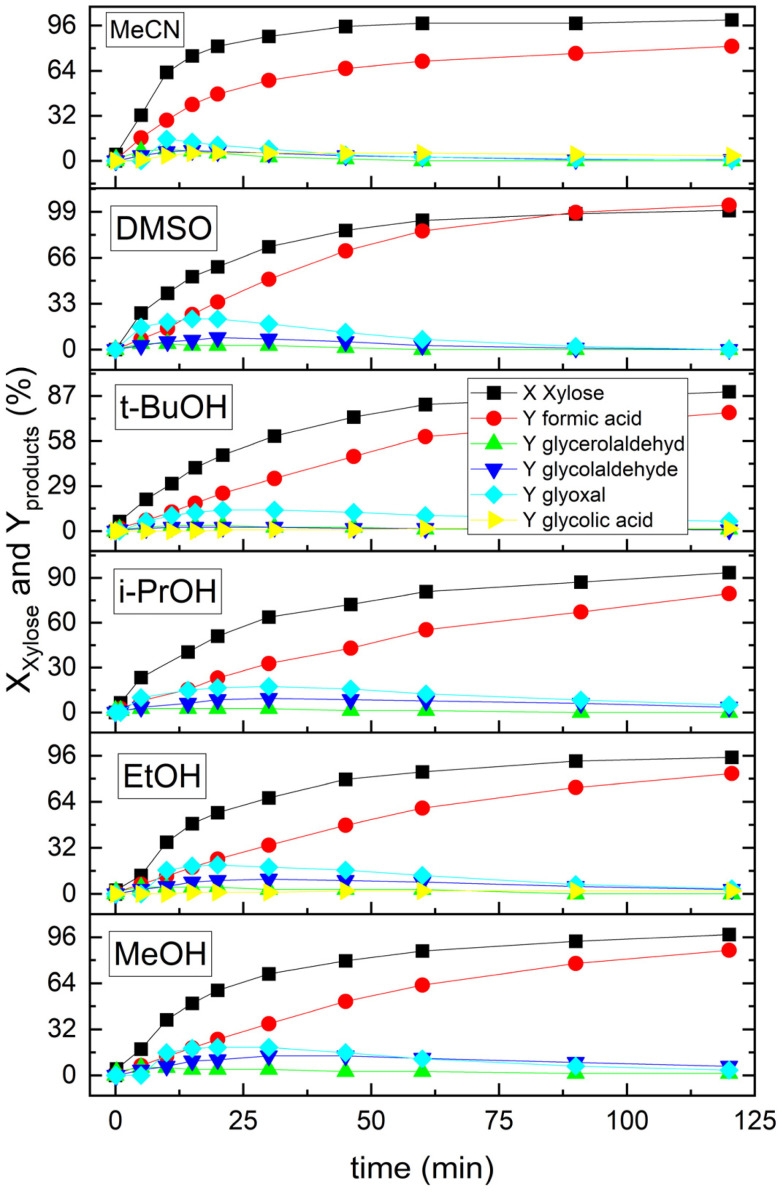
Time-resolved conversion of xylose (*X*_xylose_) and product formation (*Y*_product_) for each experiment conducted with selected co-solvents. Reaction conditions: *c*(H_8_PV_5_Mo_7_O_40_) = 15 mmol L^−1^, *c*(xylose) = 50 mmol L^−1^, *p*_O_2_, start_ = 50 bar, *V*_stirrer_ = 1000 rpm, solvent composition: 90 : 10% H_2_O:co-solvent or 100% water; *T* = 100 °C, and *V* = 45 mL.

Based on the aforementioned results, MeOH, EtOH, *i*PrOH, *t*BuOH, DMSO and MeCN were selected as suitable co-solvents for further investigation and compared to the pure aqueous system. Here, a special focus was on the influence of chosen co-solvents on the xylose oxidation reaction at different reaction temperatures (60 °C, 80 °C and 100 °C) addressing the key influences on the kinetic behaviour and possible selectivity changes. The conversion and product yields for performed experiments at 100 °C can be found in Fig. 1 (full results for 60 °C and 80 °C are shown in section 4, Tables S4–S24 in the SI). Additionally, the co-solvent stability was investigated by GC-MS (Table 2).

**Table 2:** Side-products emerging from co-solvents. Injection into GC-MS relates to thermal decomposition due to measurement conditions not related to catalysis. This was determined by injection of a stock-solution. Decomposition related to catalytic reaction was determined by measurement of the final reaction sample. (s = strong, m = medium and w = weak)

Additive	Decomposition products caused by injection into GC-MS	Decomposition products caused by catalytic reaction
MeOH	Stable	Formaldehyde (m), methylal (w)
EtOH	Stable	Acetic acid (w), acetaldehyde (m)
*i*PrOH	Stable	Acetone (m)
*t*BuOH	2-Methyl-1-propene (s)	MeOH (w)
DMSO	Dimethylsulfide (s), ethanethiol (m), methanethiol (m)	Dimethylsulfone (s), formaldehyde (m)
MeCN	Stable	Acetic acid (w), ethylene (w)

**Fig. 2:**
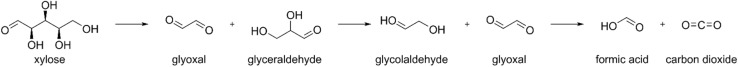
Modified reaction scheme of xylose oxidation to FA in an aqueous medium.^44^

Across all the investigated co-solvents, glyoxal was identified as the primary intermediate in the oxidation of xylose (Fig. 2). Yields of approximately 20% were achieved with MeOH, EtOH, and DMSO, whereas lower yields of around 15% were observed for all other co-solvents. Notably, MeCN and DMSO facilitated faster glyoxal conversion, indicating a reduced stabilization of the aldehyde functionality in these co-solvents. The secondary key intermediate, glycol aldehyde, also displayed a wide range of yields across the tested co-solvents, with the highest yield recorded in MeOH with 14%, followed by 11% in EtOH and 9% in *i*PrOH and DMSO. MeCN showed rapid and nearly complete conversion of this intermediate, whereas *t*BuOH exhibited minimal formation. Glycolic acid was predominantly detected with MeCN, reaching a yield of 6%, while only minor quantities (<2%) were formed in the presence of *t*BuOH and EtOH. Glycerol aldehyde was generally present in yields below 4%, except for MeCN, where a yield of approximately 8% was observed. Additionally, for DMSO, a carbon balance > 100% was detected, which can be related to degradation with the co-formation of additional FA. Overall, the reaction pathways for the chosen co-solvents (10 vol%) do not vary significantly, indicating a similar mechanistic route, as shown in Fig. 2 under the studied reaction conditions.

Additionally, stock- and end-point reaction solutions were analysed by GC-MS to obtain qualitative information on co-solvent degradation (Table 2). When injecting (inlet temperature = 250 °C) the stock-solution, thermal degradation behaviour is observable. Here, DMSO is reduced to dimethylsulfide, ethanethiol and methanethiol under measurement conditions. *t*BuOH is also dehydrated to 2-methyl-1-propene. The measurement of the end-point reaction solution shows strong oxidation of DMSO to dimethylsulfone, medium formation of formaldehyde, and medium oxidation of *i*PrOH to acetone. MeOH forms small amounts of methylal and medium amounts of formaldehyde. EtOH is slightly oxidised to acetic acid and acetaldehyde, whereas *t*BuOH only forms small amounts of MeOH. MeCN is converted to acetic acid and ethylene to a small degree. Additional analysis of reaction samples shows MeCN degradation to HCN (significant characteristic odour) and CH_4_ (gas-analysis by GC-FID); additionally, CO_2_ and ammonia (1 : 1 : 1 triplet visible in ^1^H-NMR measurements) were found.

### 2.2 Kinetic investigations of suitable co-solvents

As observed in Fig. 1, all experiments consistently demonstrate first-order kinetics in the conversion of xylose to FA (100 °C). A deeper understanding of kinetic effects can be gained by the determination of effective reaction rates (*r*_eff_) and temperature dependency by activation energy (*E*_a_) for each co-solvent.

#### 2.2.1 Comparison of effective reaction rates

**Fig. 3:**
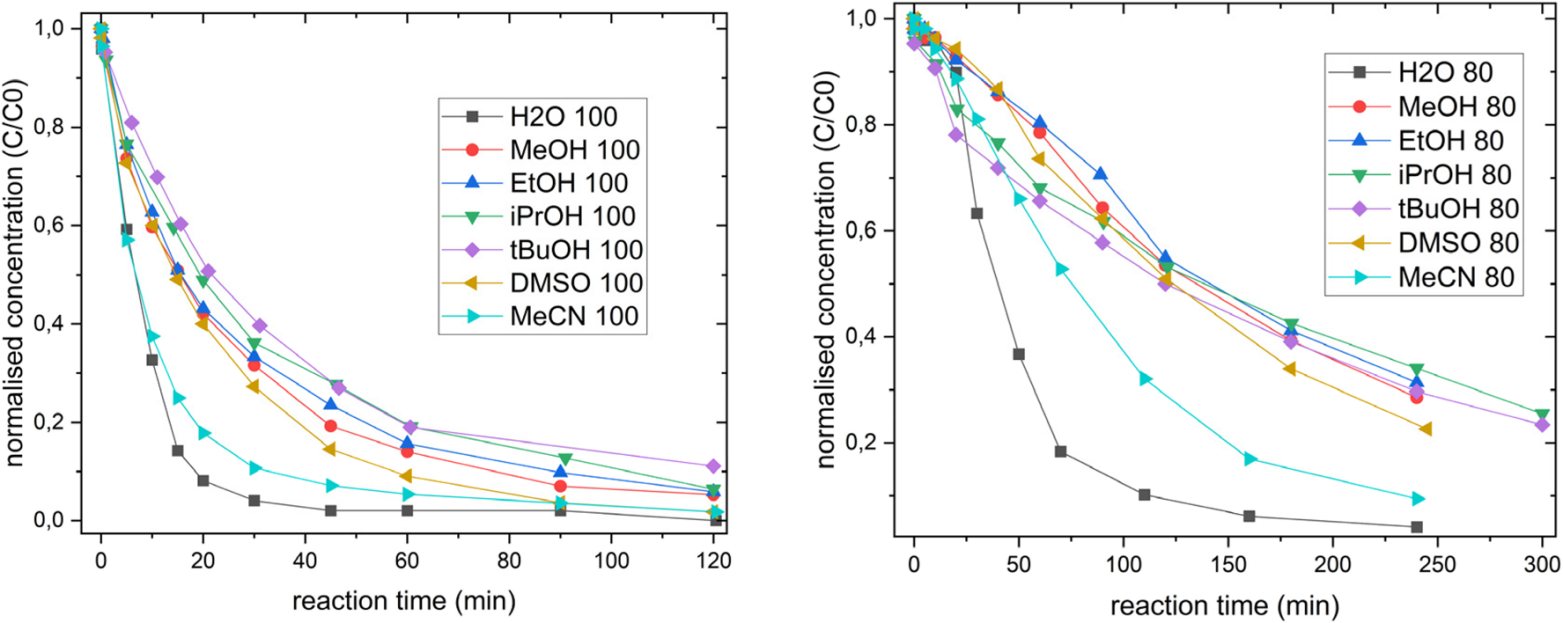
Normalised concentration of xylose *vs.* reaction time at 100 °C (left) and 80 °C (right) reaction temperature. Results for 60 °C can be found in the SI. For MeCN and H_2_O, the first ten minutes, and for all others, the first 30 min were chosen (100 °C experiments) for linearisation. Reaction conditions: *c*(H_8_PV_5_Mo_7_O_40_) = 15 mmol L^−1^, *c*(xylose) = 50 mmol L^−1^, *p*_O_2_, start_ = 50 bar, *V*_stirrer_ = 1000 rpm, solvent composition: 90 : 10% H_2_O:co-solvent or 100% water; *T* = 80 °C or 100 °C, and *V* = 45 mL.

To determine the effective reaction rates, the xylose concentration was normalized (initial concentration = 1) and plotted over reaction time (Fig. 3). Within the initial linear region, linear regression was performed for each co-solvent. For MeCN and H_2_O, the first ten minutes, and for all others, the first 30 min were chosen (100 °C experiments) for linear regression.

At 80 °C, all co-solvents exhibited an initial short induction period (Fig. 3, right). Depending on the co-solvent used, three distinct kinetic profiles can be observed. Solvent mixtures containing MeOH, EtOH or DMSO exhibited slow kinetics during the first 75 min, followed by a gradual increase in xylose conversion to approximately 80%. In contrast, solvent mixtures containing *i*PrOH or *t*BuOH exhibit rapid initial conversion within the first 25 min. Afterwards, the reaction rate decreased significantly, approaching the same conversion levels as the slower systems. These curves also display a mirrored S-shape, but reached lower final conversion. MeCN as a co-solvent showed the steepest reaction profile, achieving near-complete xylose conversion within approximately 25 min, slightly outperforming the others. These reaction profiles are characterised by a steep initial increase, reflecting sigmoidal behaviour.

At 100 °C, the reaction behaviour changes and no distinct induction periods were observed in the experiments (Fig. 3, left). Instead, two predominant trends emerged. In solvent mixtures containing MeOH, EtOH, *i*PrOH, *t*BuOH and DMSO, a xylose conversion of 50% was reached after 20 min, reaching around 80% after 40 – 60 min, with nearly full conversion (>95%) after 120 min. Conversely, the H_2_O : MeCN mixture exhibits the fastest initial kinetics, reaching at least 80% conversion within 20 min and achieving full conversion within 90 min, outperforming the other co-solvents during the initial reaction phase and approaching the performance of the pure aqueous system. Additionally, experiments at 60 °C reaction temperature were conducted with a prolonged reaction time of up to 1440 min (full results for all performed experiments can be found in Tables S4–S24). All kinetic experiments were analysed within the linear regime of each dataset, and effective reaction rates (*r*_eff_) were calculated (Table 3).

**Table 3:** Effective reaction rates for all the tested co-solvents (10 vol%) at 60 °C, 80 °C and 100 °C. Reaction conditions: *c*(H_8_PV_5_Mo_7_O_40_) = 15 mmol L^−1^, *c*(Xylose) = 50 mmol L^−1^, *p*_O_2_, start_ = 50 bar, *V*_stirrer_ = 1000 rpm, solvent composition = 90 : 10% H_2_O:co-solvent or 100% water; *T* = 60 °C, 80 °C or 100 °C, and *V* = 45 mL

Additive	H_2_O	MeOH	EtOH	*i*PrOH	*t*BuOH	DMSO	MeCN
*r*_eff_ @ 60 °C (mol L^−1^ min^−1^)	0.03	0.02	0.01	0.01	0.02	0.02	0.03
*r*_eff_ @ 80 °C (mol L^−1^ min^−1^)	0.60	0.21	0.18	0.17	0.19	0.22	0.34
*r*_eff_ @ 100 °C (mol L^−1^ min^−1^)	3.81	1.92	1.72	1.29	1.24	1.91	4.73
*Y*_FA_ @ 100 °C	65%	87%	84%	80%	76%	98%	81%
*Y*_CO_2__ @ 100 °C	35%	1.7%	2.8%	1.7%	3.2%	1.5%	12%

The reactions conducted at 60 °C exhibited *r*_eff_ values (in mol L^−1^ min^−1^) ranging from 0.013 for *i*PrOH to 0.033 for pure water and MeCN acted as co-solvents. At 80 °C, the reaction in pure water had the highest *r*_obs_ value of 0.60, which was approximately three times higher than the values observed with MeOH, EtOH, *i*PrOH, *t*BuOH and DMSO, and almost double that observed with MeCN. This enhanced rate emphasises the influence of temperature on the reaction kinetics and the potential suppressive effect of certain co-solvents. At 100 °C, MeCN exhibited the highest *r*_eff_ value of 4.73, which exceeds even the *r*_eff_ value of 3.8 of the pure aqueous solvent. The other co-solvent mixtures continued to show comparatively low rates, similar to those observed at 80 °C. Collectively, these data suggest that with each increase of approximately 20 °C, the *r*_eff_ value increases by about one order of magnitude, illustrating a strong temperature dependence of the reaction kinetics that is consistent with a classical Arrhenius-type behaviour. This trend highlights the importance of optimising temperature conditions further to achieve higher reaction rates and efficiencies for the modified OxFA process. Consequently, optimizing the reaction conditions at elevated temperatures can lead to substantial reductions in reaction time and improvements in the overall process efficiency. Additionally, it is imperative to exercise caution and avoid excessive increases in temperature, as this may lead to significant safety concerns, particularly in the context of high-pressure oxygen in combination with organic solvents, which can pose a substantial explosion risk. Therefore, great care was taken to conduct all experiments far below the lower explosion limit of each co-solvent's vapour.

#### 2.2.2 Correlation of reaction rates with oxygen solubility

Previous studies show that the reoxidation of the HPA-5 catalyst (V^4+^/V^5+^) is the rate-determining step (RDS) for biomass oxidation reactions using vanadium-substituted POM catalysts.^37,39^ Therefore, the molecular oxygen in the gas-phase needs to be dissolved into the aqueous reaction solution, where it can be used as an oxidant for the reduced V^4+^ catalyst. To relate the influence of the selected co-solvents on the solubility and possibly explain the different effective reaction rates, information on oxygen solubility is necessary. However, literature provided no data regarding the chosen co-solvents in mixtures with water (10 vol%) and at elevated pressure (*p* = 50 bar) and temperature (*T* = 100 °C).

**Fig. 4:**
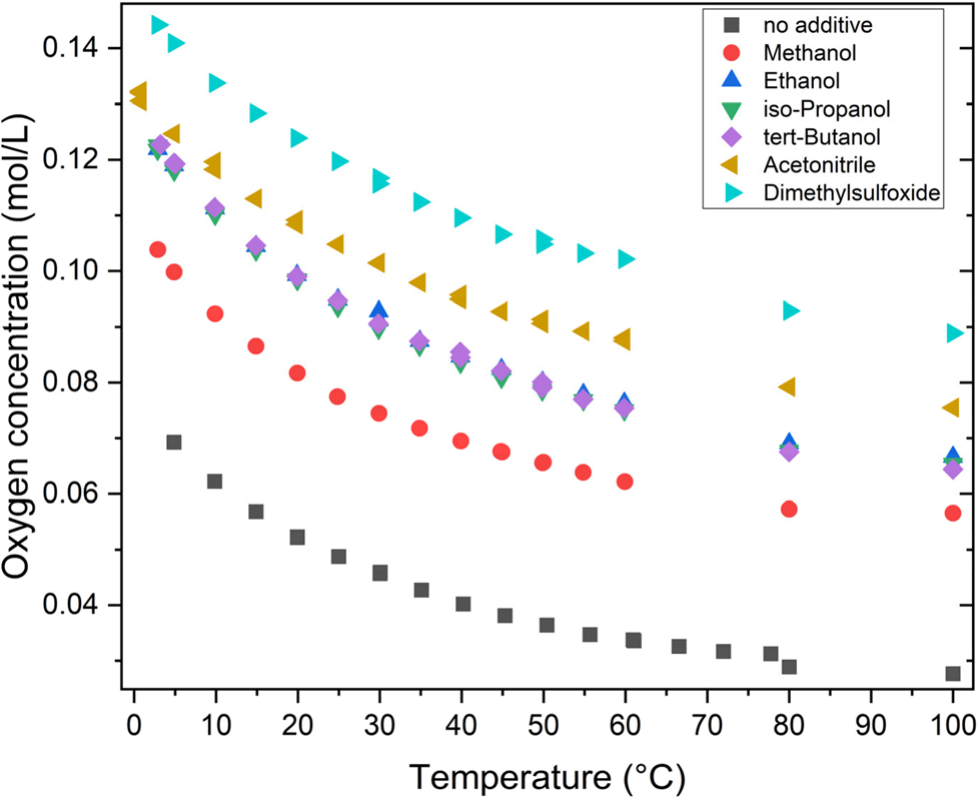
Oxygen solubility measurements for different temperatures and tested co-solvent mixtures (10 vol%). Measurement conditions: full data can be found in section 5 (Tables S31–44) of the SI for all measured co-solvents.

Oxygen solubility measurements (Fig. 4, S38 and S39) show significant differences for the various co-solvents used. Note that measurements for organic co-solvents were limited to *T* = 60 °C due to the corrosive behaviour of pressurized oxygen. As expected and well documented in the literature, the solubility of oxygen in pure water is inherently low.^50^ Initial measured values are around 69 mmol L^−1^ O_2_ at 5 °C, decreasing to approximately 34 mmol L^−1^ O_2_ at 60 °C. Adding 10 vol% MeOH effectively doubles oxygen solubility at 60 °C, reaching 62 mmol L^−1^ O_2_. Similarly, minor increases in oxygen solubility were observed with the addition of polar protic solvents (10 vol%), such as EtOH, *i*PrOH and *t*BuOH. The measured values were approximately 76 mmol L^−1^ at 60 °C, which is consistent with their ability to facilitate the dissolution of gases through hydrogen bonding and polarity effects. In contrast, solvent mixtures with polar aprotic solvents such as MeCN and DMSO exhibit significantly higher oxygen solubilities, with measured values of 87 mmol L^−1^ and 102 mmol L^−1^ at 60 °C, respectively (Fig. 4). These findings indicate that aprotic polar solvents often enhance gas solubility due to their strong dielectric constants and ability to stabilise dissolved oxygen molecules without interference from hydrogen bonding.

**Fig. 5:**
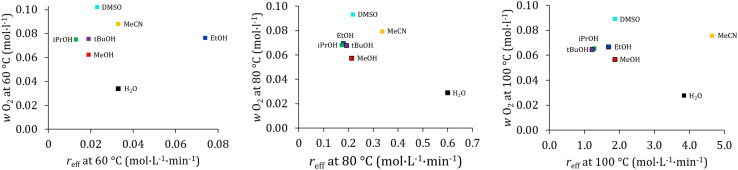
Oxygen solubility *vs. r*_eff_ at 60 °C (left), 80 °C (middle) and 100 °C (right). Measurement conditions: full data can be found in Tables S31–S44 in the SI for all measured co-solvents.

When gas solubilities in different solvents as a function of reaction rate are evaluated (Fig. 5), it becomes apparent that increased oxygen solubility does not necessarily lead to an increased reaction rate.

As reaction dynamics are governed not only by gas solubility but also by factors such as solvent polarity, hydrogen-bonding capacity, and the catalytic environment, comparisons across all solvent mixtures are challenging. A clearer trend emerges when focusing on water, MeOH, EtOH, *i*PrOH, and *t*BuOH. These solvent mixtures can be considered chemically similar: each contains a hydroxyl group bound to an increasing number of carbon atoms, starting with water (zero carbon atoms), followed by MeOH (one carbon atom), EtOH (two carbon atoms), and so forth. The most pronounced trend is observed at 80 °C. Water, with the lowest number of carbon atoms, exhibits the lowest oxygen solubility and the highest reaction rate. MeOH, with the second-lowest carbon number, shows the second-highest reaction rate. EtOH, *i*PrOH, and *t*BuOH display nearly identical reaction rates and more or less identical oxygen solubilities. The same trend is observed at 60 °C and 100 °C, with minor deviations attributable to experimental uncertainties. Therefore, within this subset of polar protic co-solvents, a consistent relationship is evident: lower oxygen solubility correlates with a higher reaction rate. At first glance, this is counterintuitive. One might expect that higher oxygen concentrations would accelerate catalyst reoxidation. However, increased oxygen solubility arises from stronger interactions between the solvent and oxygen molecules. Consequently, oxygen may be less available for catalyst reoxidation because the alcohol competes with the catalyst for access to dissolved oxygen. In contrast to the alcohols, the polar aprotic co-solvents DMSO and MeCN do not fully follow this trend. It should be noted, however, that the mass fraction of DMSO in water was nearly 11 percent, whereas the mass fractions of the alcohols and acetonitrile were 8 percent. Therefore, it is more realistic to assume that DMSO at an 8-percent fraction would exert approximately the same influence on solubility as the alcohols. As its reaction rate is also similar to that of the alcohols, DMSO shows the same relationship between oxygen solubility and reaction rate, as observed for the alcohols.

#### 2.2.3 Correlation of reaction rates with the redox potential

**Fig. 6:**
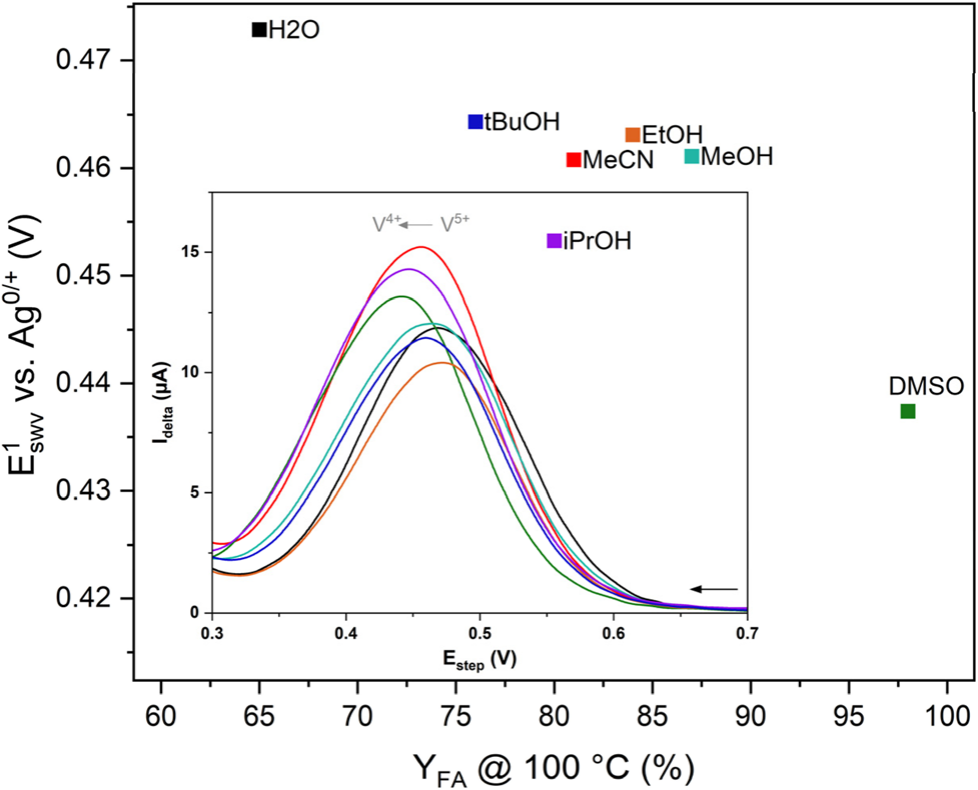
Correlation between the first reduction of HPA-5 in different solvent mixtures (90% H_2_O and 10% of the indicated co-solvent) as determined by SWV and FA-yield. Inset*:* SWV investigation of the first redox event [Concentration: 1 mmol L^−1^, scan rate: 5 mv s^−1^, and pH 1 (0.1 M hydrochloric acid was used as the supporting electrolyte.)]. Data were smoothed using fast Fourier transformation to eliminate high-frequency noise. The raw data are shown in section 2.4 in the SI.

To gain deeper insights into the principles governing catalytic performance enhancements induced by different co-solvents, the electrochemical behaviour of HPA-5 was investigated using CV (Fig. S5–S13) and SWV (Fig. S14 and S15) for the seven solvent mixtures described above under acidic conditions. Consistent with previous studies on aqueous HPA-5 solutions,^28,39,45^ the electrochemical profiles reveal a complex pattern of multiple, partially overlapping redox events corresponding to both irreversible reductions of molybdenum centres within the polyoxomolybdate framework and reversible vanadium-centred reductions under nitrogen atmosphere (Fig. 6).

Since the primary interest lies in catalytic activity, a focus was on the first reduction event (Fig. 6, inset). It is hypothesized that the ease of xylose oxidation is directly linked to the redox potential observed in different co-solvent mixtures. Indeed, a correlation between the redox potential determined by SWV and the effective reaction rate (*r*_eff_) obtained from catalytic reactions at 100 °C was established (Fig. 6). In general, higher redox potentials for this first event correlate with lower FA-yields (*e.g.*, pure H_2_O: 0.473 mV, 65% *Y*_FA_, H_2_O/*t*BuOH: 0.464 mV, 76% *Y*_FA_), supporting the initial hypothesis that thermodynamically favoured electron transfer processes enhance product formation (DMSO needs to be excluded due to its own decomposition towards FA). However, *i*PrOH represents an exception to this trend. Although it shows the second lowest redox potential [E^1^(*i*PrOH) = 0.447 V *vs.* Ag/AgCl; for comparison E^1^(H_2_O) = 0.468 V *vs.* Ag/AgCl], only a moderate yield of FA was obtained under these conditions. This observation points to the complexity of the reaction sequence: while the addition of *i*PrOH increases oxygen solubility (Fig. 5) and significantly lowers the redox potential (Fig. 6), solvent decomposition appears to compete with the conversion of xylose to FA. These findings emphasize that solvent effects extend beyond simple thermodynamic considerations, influencing both reaction pathways and catalyst integrity. Further *in silico* studies are therefore carried out to clarify how the co-solvents interact with the catalyst.

#### 2.2.4 Correlation of reaction rates with catalyst-solvent interactions by DFT calculations

In order to reduce the computational model to manageable complexity, H_5_PV_2_Mo_10_O_40_ (HPA-2) was used as a model POM-catalyst. Its comparability to HPA-5 has been sufficiently established in previous studies, to draw valid conclusions.^35,44,54^

To determine the most likely sites for the reduction of POMs with the different co-solvents, the model reduction reactions were calculated (Table 4). The absolute values of the reduction potentials are not important here (as these reactions are not expected to occur in the experimental system); more important are the relative reduction values when the co-solvents are bound either at the vanadium site or at a molybdenum site at the opposite end of the POM. In all cases, reduction is strongly favoured at the vanadium site, which consistently has a much lower Δ*G* than reduction at the molybdenum site.

**Table 4:** DFT-calculated reduction potentials at the vanadium sites and the molybdenum sites (opposite end of the POM from V) in the presence of each of the co-solvents

Additive	Reaction	ΔG/kJ mol^−1^	
		V	Mo
MeOH	POM + MeOH → POMH_2_ + Me = O	51.1^a^	115.6^a^
EtOH	POM + EtOH → POMH_2_ + Et = O	19.8	81.4
*i*PrOH	POM + *i*PrOH → POMH_2_ + *i*Pr = O	−1.5	63.7
*t*BuOH	POM + *t*BuOH → POMH_2_ + *t*Bu = O	−35.1	25.7
MeCN	POM + H_2_ → POMH_2_ (with MeCN bound)	−32.2	27.8
DMSO	POM + H_2_ → POMH_2_ (with DMSO bound)	−33.1	21.1

a Taken from ref. 44.

The top five binding positions (and energies) are given in Table 5. The three alcohols have similar binding energies to MeOH (all *via* hydrogen bonding), although the favoured binding position for EtOH is at the opposite end of the POM from the vanadium binding site. The binding energy of EtOH at the vanadium site is ∼35 kJ mol^−1^, and therefore, it is likely to be competitive with xylose. For *i*PrOH and *t*BuOH, the binding energy at the vanadium sites is ∼ −39.5 kJ mol^−1^ in both cases, which is also likely to be competitive with xylose. Much like the trends seen for MeOH, one would expect this to slow the effective reaction rate as the interchange of the solvent and xylose must occur.

**Table 5:** Top five binding sites for each co-solvent, with the binding free energies in parentheses (kJ mol^−1^)

Rank	EtOH	*i*PrOH	*t*BuOH	MeCN	DMSO
1	19 (−38.2)	14 (−39.7)	15 (−41.6)	1 (−23.8)	20 (−29.6)
2	3 (−35.4)	1 (−39.5)	3 (−40.2)	19 (−23.4)	14 (−29.5)
3	16 (−35.1)	15 (−38.9)	1 (−39.6)	3 (−22.8)	12 (−29.0)
4	14 (−34.4)	8 (−37.9)	12 (−38.8)	18 (−22.8)	21 (−28.9)
5	5 (−34.2)	3 (−37.5)	17 (−37.7)	5 (−22.2)	18 (−28.6)

**Fig. 7:**
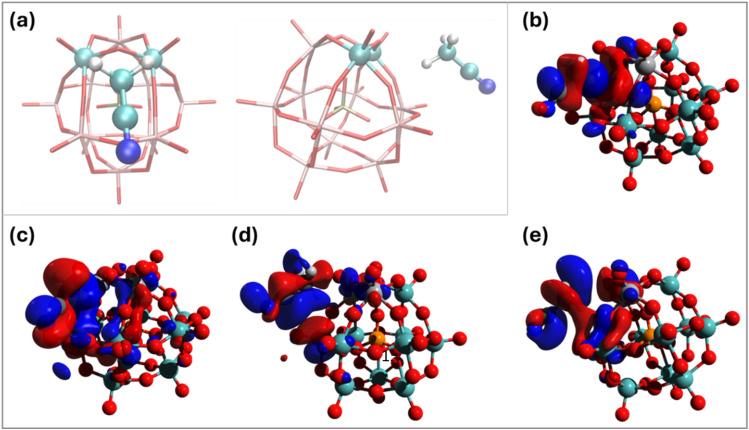
(a) Binding coordination of HPA-2 and MeCN; front view (left); side view (right). (b)–(e) Four most strongly interacting ETS-NOCV orbital pairs.

For DMSO and MeCN, the picture is somewhat different. DMSO has stronger binding energies than water and is therefore likely to displace water bound to the POM, but the preferred binding sites are far from the vanadium sites, where xylose binding is much more favourable (by ∼10 kJ mol^−1^). Guo *et al.* have previously attributed the selectivity and rate enhancement (of glucose oxidation) to the formation of hydrogen bonds between DMSO and key intermediates in the reaction,^37^ which is likely to be the case here, too. The binding of MeCN across the POM is likely to be competitive with water (whose binding energies range from −19.0 to −26.6 kJ mol^−1^); the preferred binding site is at the vanadium centres, although xylose binding is more favourable at this site. As hydrogen bonding is not responsible for the binding of MeCN and the POM, an extended transition state–natural orbitals for chemical valence (ETS-NOCV)^53^ analysis was performed to understand the nature of the interaction (Fig. 7). A series of weak interactions between the orbitals of *e* symmetry of MeCN and the O lone-pair orbitals (and overlaps with the d orbitals of predominantly V, but some Mo d orbitals) were found, essentially demonstrating non-covalent interactions similar to π–π interactions.

For both DMSO and MeCN, xylose binds more strongly to the POM when the co-solvent is also bound (by −1.9 and −2.6 kJ mol^−1^, respectively, to give total binding free energies of −53.2 and −53.8 kJ mol^−1^) than when just xylose is bound (with a binding free energy of −51.3 kJ mol^−1^). For each of the alcoholic co-solvents considered, the binding free energy decreases by > 5.4 kJ mol^−1^. While these binding free energy changes are relatively small, crucially they represent a positive change in binding in the presence of MeCN or DMSO, while xylose binds less strongly in the presence of the alcoholic co-solvents. The increased binding strength of xylose at the vanadium site is likely to lead to more efficient C–C bond breaking.

#### 2.2.5 Process intensification for the most promising co-solvent using DoE

MeCN was selected as the most suitable co-solvent as it showed the fastest reaction kinetics of all co-solvents even outperforming the aqueous reference system at high temperatures. Moreover, it demonstrated a significant selectivity increase compared to the aqueous reference system. Although MeOH showed the highest FA-selectivities, it drastically slowed down reaction kinetics and showed only a moderate stability under the applied reaction conditions.

In order to improve the overall xylose oxidation process, the next step was to optimize the reaction parameters, specifically reaction temperature, O_2_ pressure, substrate concentration and amount of co-solvent.

To draw meaningful conclusions from this investigation in an STR, the effects of film diffusion needed to be excluded first. This was tested by varying the stirrer speed (Fig. S30) at the highest xylose concentration within the DoE parameters (Table S25). The stirrer speed shows no influence on the kinetic parameters, neither on xylose conversion, nor on FA-yield or selectivity. A slight variance can be observed for the intermediate glycolic acid. At 500 rpm, the proportion of glycolic acid is the highest. All other results are the same for 1000 and 1500 rpm, therefore 1000 rpm was chosen for further experiments.

#### 2.2.6 Sensitivity analysis and parameter optimization using DoE

A Central Composite Design (CCD) was chosen for the optimization of the process parameters towards a maximized FA-yield as it covers a wide parameter range and has more flexibility regarding boundary conditions than the other response surface designs (RSF) for increasing the economic efficiency of the modified OxFA process. Herein, the influence of the four parameters, namely (A) reaction temperature between 90 and 150 °C, (B) partial oxygen pressure between 5 and 50 bar, (C) substrate concentration between 50 and 350 mmol L^−1^ and (D) MeCN volume fraction between 1 and 50 vol%, was investigated at 5 levels (Tables S25). The summarized results for the responses *Y*_FA_, *X*_xylose_ and *Y*_CO_2__ can be found in Table S26.

After performing the experiments, the CCD design was evaluated statistically for each response performed, yielding three different models (see fit summary for *Y*_FA_, *X*_xylose_ and *Y*_CO_2__ in Table S27). *Y*_FA_ and *Y*_CO_2__ can be described best with a quadratic model, whereas xylose conversion is best described with a reduced two-factor (2FI) model. Even though the quadratic model for *X*_Xylose_ shows higher *R*^2^ values, the 2FI model was chosen due to the lowest sequential *p*-value.

According to the different *p*-values of each factor, a combination was chosen for implementation into the model. The results of the analysis of variance (ANOVA) can be found in Tables S28 for *Y*_FA_, Table S29 for *X*_Xylose,_ and Table S30 for *Y*_CO_2__, respectively.

**Fig. 8:**
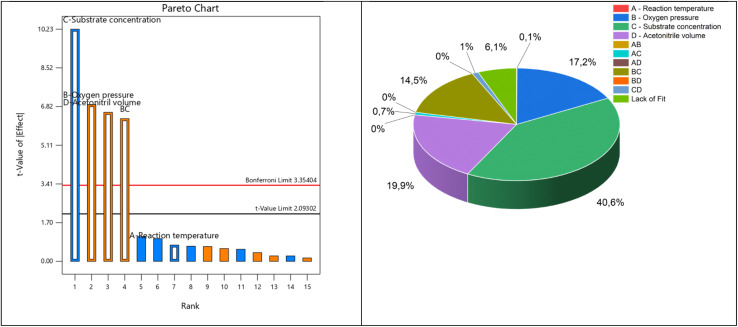
Pareto Chart for the integration of the RS model in the FF design (left) and the proportion of influencing parameters in the statistical design of experiment (right).

The parameters B (*p*O_2_), C (*c*_Xylose_) and D (MeCN vol%) have the most significant influence on the response *Y*_FA_, yet A (temperature) is significant with a *p*-value < 0.05. Additionally, the parameter combinations AB, AD and BD are not significant, but included within the model to maintain hierarchy. The predicted *vs.* experimental graph for *Y*_FA_ (Fig. S31) shows a high agreement of the model with the experimental data. Furthermore, the experimental design and the results can be integrated within a full factorial (FF) model to validate the obtained data. Therefore, a regular two-level factorial design with 16 runs was extended with 11 additional runs. A Pareto Chart was drawn to analyse, select and include the most significant factors within the model (Fig. 8).

The parameter C (substrate concentration) has a negative influence on *Y*_FA_; however, a positive influence is observed by the other significant parameters B, D and BC. The reaction temperature is below the Bonferroni Limit (3.35) and the *t*-value limit (2.09) but included within the model to maintain hierarchy. Though the resulting model is not orthogonal (hence its origin from RSM), an adjusted *R*^2^ value of 0.901 could be obtained. The main parameters B, C and D alone contribute to 77% of the model for *Y*_FA_ response. The lack of fit has only an influence of 6%, showing a valid approach for describing the experiments. For optimization, the same parameters as for the CCD design were chosen. The optimization suggests *Y*_FA_ of 89%, *X*_Xylose_ of 99% and *Y*_CO_2__ of 4.2% at *T* = 126 °C, *p*_O_2__ = 43 bar, *c*_Xylose_ = 101 mmol L^−1^ and 42 vol% MeCN as the co-solvent in water. Therefore, both the models show similar results regarding optimum reaction conditions and resulting yields. Consequently, the experimental verification test was performed three times (*n* = 3) with those parameters yielding *Y*_FA_ of 90% (±1.11%), *X*_Xylose_ of 100% (±0%) and *Y*_CO_2__ of 4.83% (±0.35%).

**Fig. 9:**
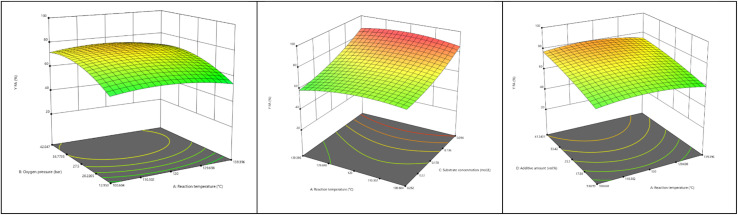
Resulting 3D surface plots for the formic acid yield. Parameters A and B (left), C and A (middle) and D and A (right).

The model is able to describe 99.5% of the experimental data. As discussed above, for converting xylose, the co-solvent amount shows no significant influence and is therefore not contained within the model. Here, the oxygen pressure and the correlation between *p*_O2_ and the substrate concentration are of highest significance (*p* = 0.006). The parameters AB and AC are included showing less significance (*p* = 0.015), yielding an *R*^2^ value of 0.649 for response *X*_Xylose_. The ANOVA for the response *Y*_CO_2__ yields a quadratic model, where the reaction temperature (*Y*) shows the lowest significance of the chosen main parameters (*p* = 0.031). The combined factors AB, AC and AD show *p* values taller *p* = 0.05, but were included to maintain hierarchy. The most significant influence is shown by parameters B (partial oxygen pressure) and D (MeCN volume fraction). The derived model is able to describe 97.3% of the experimental data. The resulting 3D surface plots for *Y*_FA_ as response can be found in Fig. 9.

The highest influence of a parameter set can be observed for the combination of A (temperature) and C (substrate concentration). The FA yield ranges from 58% to 92%, showing a broad range of results; the higher the substrate concentration is, the less the FA produced. The reaction temperature has a sweet spot at around 115 °C. As seen within the ANOVA, the parameter combination A and B, as well as A and D, has less influence on *Y*_FA_, in general higher oxygen pressures lead to higher *Y*_FA_ – and higher additive volume shows the same trend for *Y*_FA_.

As deduced from oxygen solubility measurements, higher temperatures (A) lower the solubility of oxygen in the solvent mixture (Fig. 4). However, higher partial oxygen pressures (B) and higher additive amounts (D) lead to increased oxygen solubility. This shows two opposing trends, which lead to a non-linear behaviour resulting in a “sweet spot”. This fits the experimental trends well: a low partial oxygen pressure leads to high xylose conversion, but *Y*_FA_ is low due to the high amount of remaining intermediates. This trend is also true for an increased substrate amount. Additionally, a combination of both low oxygen pressure and high substrate amount at high reaction temperatures leads to a by-product-rich reaction solution containing furfurals and humin-like solids, whereas the catalyst is not reoxidized, as indicated by the blue colour of the resulting reaction solution. When observing the results for *X*_Xylose_, a dominant number of experiments resulted in conversion values *X* > 97%. Parameters B and D show the biggest influence yielding results from *Y*_CO_2__ of 2–19%, showing a very broad range. The highest values are reached at high substrate loads, high partial oxygen pressures and low co-solvent amounts. This shows the significance of adding a minimum amount of co-solvents for the inhibition of CO_2_ formation.

## 3 Conclusion

The beneficial use of co-solvents in the modified OxFA process (10 vol% co-solvent) was evaluated using a combination of spectroscopic (NMR, UV-Vis, and GC-MS), electrochemical (CV and SWV), and thermodynamic (gas solubility measurements) investigations in combination with theoretical modelling of the catalyst–solvent interactions by DFT. Hereby, a special focus was on the influence of system stability, reaction rates and kinetic performance parameters for the HPA-5 catalyzed oxidation of xylose to formic acid. In detail, the pure aqueous system maintains the second highest reaction rate paired with the lowest formic acid selectivity of 66%. The addition of polar protic solvents like alcohols (MeOH, EtOH, *i*PrOH and *t*BuOH) significantly decreases reaction rates at all investigated reaction temperatures while improving the formic acid selectivity (>80%). The reaction rate was also low for the polar aprotic DMSO added as a co-solvent, whereas its excessively high FA yield can be related to significant DMSO degradation. Oxygen solubility measurements showed a strong difference between the investigated co-solvents. DMSO exhibits the highest oxygen solubility, whereas MeOH has the lowest values for all co-solvents; yet, this improvement has not been reflected in enhanced reaction rates. Interestingly, a clear trend for formic acid yield *vs.* redox potential was found, where higher values in redox potential lead to decreased FA yields.

MeCN seems to be the most promising co-solvent; high oxygen solubility values and lower catalyst redox potential in MeCN/H_2_O mixtures resulted in the highest reaction rates with high FA yields, using reaction parameters that differ from the state of the art. Moreover, the high temperature dependency shown by the Arrhenius plot offers an intensification of the process by optimising the reaction conditions, increasing the productivity even further. Additionally, the volume of the co-solvent could improve the reaction (with being inside a safe limit regarding lower explosion limit) regarding selectivity and yield of formic acid. To examine whether a lower oxygen partial pressure is suitable for reducing costs of compression and plant design, a variation needs to be performed. With the increased substrate concentrations, higher amounts of FA can be produced with improved economy of the process. Overall, these findings comprise a significant step for the improvement of the OxFA process, thereby contributing to a more efficient sustainable production of formic acid.

## 4 Experimental section

### 4.1 Materials and catalyst synthesis

All chemicals were obtained commercially and used as received without further purification. d(+)-Xylose (99%), glyoxal (40%) and glyceraldehyde (90%) were supplied by Merck KGaA, glycolic acid (99%) was bought from Acros Organics and glycol aldehyde-dimer (99%) was obtained from Sigma-Aldrich; the solvents methanol (MeOH, 99.8%), ethanol (EtOH, 99.8%) and acetonitrile (MeCN, 99.9%) were bought from VWR chemicals, *n*-propanol (*n*-PrOH, 99%), *iso*-Propanol (*i*-PrOH, 99%), *n*-butanol (*n*-BtOH, 99%), *s*-butanol (*s*-BtOH, 99%), *t*-butanol (*t*-BtOH, 99%), dimethyl sulfoxide (DMSO, 99.5%), ethylene glycol (99%) and butanone (99%) were supplied by Gruessing GmbH, and acetone (99.99%) was bought from Fischer Scientific. Demineralized water (DI) was used as the main solvent. For carrying out the catalytic experiments, oxygen (5.0 quality) was bought from Linde AG.

The vanadium-substituted H_8_PV_5_Mo_7_O_40_ (HPA-5) catalyst was kindly provided by OxFA GmbH. The characterization of the catalyst was carried out using a Fa. Spectro Arcos ICP-OES device resulting in a P/V/Mo ratio of 1.27/4.78/7.00. The Keggin-structure type was verified by FT-IR spectroscopy using an IRSpirit-X equipped with an ATR unit from Shimadzu, showing the typical stretching vibrations associated for the Keggin oxo-anions of P–O_*a*_, Mo = O_*d*_, Mo–O_*b*_–Mo, and M–O_*b*_–Mo bonds that were detected at 1045, 948, 869, and 739 cm^−1^, respectively, where *Y*_b_ refers to the oxygen atom that connects the two trimetallic groups, O_*c*_ joins the two octahedral MoO_6_ units inside the trimetallic group, O_*d*_ is the terminal oxygen atom, and O_*a*_ is the oxygen atom connecting the PO_4_ unit of the tetrahedron and the trimetallic Mo_3_O_13_ group. The crystal water content was determined to be 12H_2_O per H_8_PV_5_MO_7_O_40_ by thermogravimetric analysis (TGA) using a TG 209 F1 Libra from Netsch at a heating ramp of 15 K min^−1^ to 350 °C.

### 4.2 Experimental procedure

All kinetic experiments were conducted in a 3-fold high-pressure screening plant (Fig. S1) in a batch operation mode. The plant has already been described in detail in the literature.^44^

In a typical experiment, glass liners were filled with 45 mL of a reaction solution, which was prepared with 1.25 g (15 mmol L^−1^) catalyst (HPA-5), 0.38 g (50 mmol L^−1^) substrate (xylose) and 50 mL of solvent. Here, either 100 vol% H_2_O or a mixture of 90 : 10 vol% H_2_O:co-solvent was used. The filled glass liners were inserted into the autoclaves. After closing with the appropriate torque ensuring leak tightness, the autoclaves were purged three times with 35 bar oxygen to ensure a pure oxygen atmosphere. For experiments at a reaction temperature of 100 °C, the autoclaves were pressurized to an initial pressure of 45 bar at ambient temperature. Subsequently, the desired reaction temperature and a stirrer speed of 300 rpm were set. When the inside temperature reached the desired value, the zero-minute sample was drawn from the sampling valve. The stirrer speed was subsequently increased to 1000 rpm starting gas-entrainment. The samples were drawn after shown reaction times and directly put on ice. When the reaction was finished, the stirrer speed was decreased to 300 rpm, the heating jackets were taken off and the reactors were cooled with pressurized air. After cooling the reactors to room temperature, samples of the gas phase were taken, and subsequently, the autoclaves were vented, and further analysis of the reaction solutions was carried out.

### 4.3 Further sample preparation

Directly after the reactions performed at 100 °C, *ex situ* UV-Vis spectroscopy was performed using a Cary 60 Spectrophotometer by Agilent. For this purpose, the reaction samples were taken from the reactor and measured non-diluted in 1 mL cuvettes within 1100 – 500 nm wavelength. Afterwards, the samples from these reaction solutions were prepared for Nuclear Magnetic Resonance (NMR) spectroscopy as well as High-Performance Liquid Chromatography (HPLC) and Gas chromatography coupled with mass spectrometry (GC-MS). The catalyst was analysed further by ^51^V and ^31^P-NMR spectroscopy using a Bruker Avance III HD 600 MHz spectrometer (base frequency: 600.13 MHz) equipped with a 5 mm BBFO smart probe. All other experiments were investigated by using HPLC and ^1^H-NMR spectroscopy.

### 4.4 Determination of the quantitative reaction parameters

After the reaction, the pH values of all mixtures were measured using a pH probe (Fig. S2). All reaction products were quantitatively determined by HPLC, NMR- and GC- (Gas Chromatography) analysis. Liquid-phase analysis was performed using HPLC and ^1^H-NMR spectroscopy. An exemplary HPLC-Chromatogram can be found in Fig. S3 and the GC-MS chromatograms of all the stock solutions in Fig. S4. For NMR-analysis, 500 µL reaction solution was mixed with either 75 µL D_2_O–10 wt% *t*BuOH or a similar acetone (replacing *t*BuOH) solution. The conversion of substrates and yields of all liquid products were determined by means of HPLC measurements using a Nexera-40 HPLC from Shimadzu equipped with a 300 mm × 8 mm organic acid column from CS-Chromatographie Service GmbH and a refractive index detector. Then, 4 mmol L^−1^ of an aqueous sulfuric acid solution was used as the eluent and the samples were filtrated before analysis through a syringe filter (45 µm). The yields of FA and the corresponding derivatives were quantified by ^1^H-NMR using a Bruker Avance III HD 600 MHz spectrometer. Where applicable, the xylose concentration was measured by ^1^H-NMR spectroscopy. The determination of the gaseous by-products CO_2_ and CO was done by means of GC analysis using a Varian GC 450 equipped with a 2 m × 0.75 mm ID ShinCarbon ST column. No other gaseous products could be detected by the used GC.

### 4.5 Electrochemical investigations

All the electrochemical measurements were performed in an aqueous hydrochloric acid solution at pH 1, with an analyte concentration of 1 mmol L^−1^, using a BioLogic SP-150e potentiostat (VMP3B-5 chassis). Prior to each experiment, the electrolyte solution was thoroughly purged with nitrogen gas to remove dissolved oxygen. The electrochemical setup consisted of a 3 mL cell equipped with a conventional three-electrode configuration: a glassy carbon working electrode (3 mm diameter), a platinum wire counter electrode, and an Ag/AgCl reference electrode. Prior to each measurement in a new solvent mixture, the working electrode was regenerated using standard procedures, including rinsing with water, polishing with 0.05 µm alumina slurry on polishing pads, subsequent washing with Milli-Q water, and drying.

Cyclic voltammetry (CV) was recorded over a potential window from +1.0 V to −0.6 V (*vs.* Ag/AgCl) at a scan rate of 100 mV s^−1^ for three consecutive scans. The measurement direction is shown in Fig. S5–S13. Square-wave voltammetry (SWV) was carried out over the same potential window with instrument settings of PH = 20 mV, PW = 40 ms, and SH = 0.4 mV, corresponding to a modulation amplitude of 20 mV, a frequency of 12.5 Hz, and a scan rate of 5 mV s^−1^. These electrochemical parameters were selected as a compromise between spectral resolution and measurement sensitivity, ensuring reliable detection of overlapping redox events (Fig. S14–15 and Table S1).

### 4.6 Calculations

The substrate conversion *X*_xylose_ was determined from the HPLC results of the liquid phase according to eqn (1), assuming constant volume:1
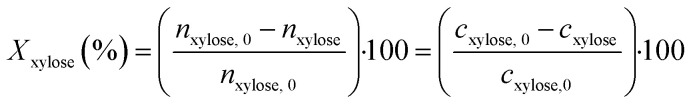
In eqn (1), *n*_xylose, 0_ is the amount and *c*_xylose, 0_ the concentration of xylose at time zero before the start of the reaction and *n*_xylose_ is the amount and *c*_xylose_ the concentration of xylose at the respective time during or after the reaction.

With the determined amount of substance of the gas phase by GC and the measured amount of substance of the liquid phase by HPLC, the respective yield *Y*_*i*_ can be determined according to eqn (2):2
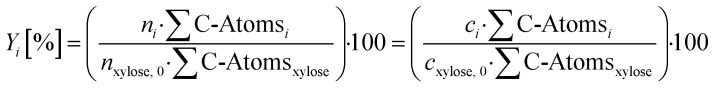
In eqn (2), ∑C-Atoms_*i*_ is the sum of the C-atoms of product *i*, ∑C-Atoms_xylose_ is the sum of the C-atoms of xylose, *n*_*i*_ is the amount and *c*_*i*_ is the concentration of product *i*.

The selectivity *S*_*i*_ of each product i is determined from the ratio of the yield *Y*_*i*_ of product i to the conversion of xylose *X*_xylose_. This is given by eqn (3):3
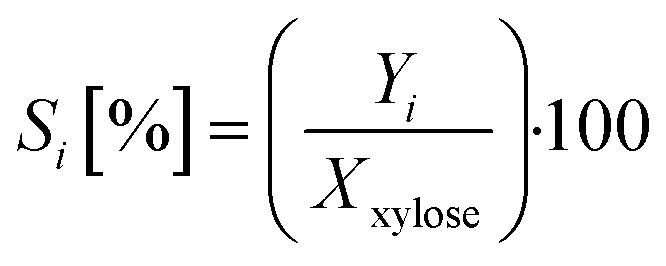


As it has been previously discussed in the literature, the observed reaction rates *r*_obs_ can be calculated using eqn (4):^21^4
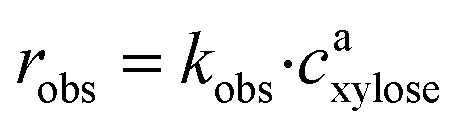
*k*_obs_ is the slope of the linear fit and *c*_xylose_ is the substrate concentration.

The determination of the activation energy (*E*_a_) was performed by plotting *k*_obs_ according to the logarithmic Arrhenius function shown in eqn (5). The slope from linearisation was divided by the ideal gas constant (*R*) yielding *E*_a_.5
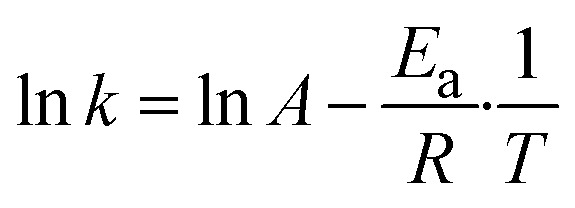


### 5.1 Design of experiments (DoE)

The DoE was planned and examined using the Design Expert 12 © software package from Stat Ease. From the initial experiments, the following parameters were fixed: *p*_total_ = 50 bar, *c*_catal*y*st_ = 15 mmol L^−1^, *V*_reaction solution_ = 45 mL, *v*_stirrer_ = 1000 rpm and reaction time (*t*) = 120 min. As responses, we focused on the xylose conversion (*X*_xylose_), FA-yield (*Y*_FA_) and CO_2_-yield (*Y*_CO_2__). For parameter optimization, the reaction temperature was varied between 90 and 150 °C, partial oxygen pressure at a fixed total pressure *p*_O_2__ between 5 and 50 bar, xylose concentration between 50 and 350 mmol L^−1^ and the partition of co-solvent between 1 and 50 vol%. With those high volumes of organic co-solvents in combination with pressurized oxygen at elevated temperatures, we ensured that the calculated reaction conditions were within the safety limits far below the lower explosion limit of the chosen solvent composition. We chose a central composite design (CCD) containing 27 runs and 3 centre points (Fig. S29). The resulting experimental plan can be found in Table S25.

### 5.2 Determination of standard deviation

For the evaluation of significance, the validation experiment of the central composite design was performed three times (*n* = 3). We tested our analytics and found standard deviations for HPLC of 0.98% for the FA yield and of 0.01% for xylose conversion.

### 5.3 Gas solubility measurements

The gas solubility was determined using the isochoric pressure drop method. The detailed description of the experimental setup and gas solubility determination procedure is given in an earlier publication.^47^ The setup consists of two parts: isothermal gas reservoir and stainless-steel measuring cell. The volume of the measuring cell (*V*_mc_) and the volume of the gas reservoir (*V*_gr_) were determined before conducting the experiments by using water and hydrogen and nitrogen expansion procedure.

The cleaned and dried measuring cell was connected to the measuring system, filled with a known amount of sample and evacuated. At the same time, the gas cylinder was filled with high-purity oxygen and thermostated at 303.15 ± 0.03 K. After achieving the isothermal and isobaric stabilization of the gas cylinder, the valve separating the two parts (gas reservoir and measuring cell) was opened allowing the gas to contact the liquid. The equilibrium between gas and liquid was assumed to be reached when the gas pressure in the system remained constant over 1 hour within the sensitivity of the pressure sensor (1 mbar). After that, the temperature of the measuring cell was changed to the next setpoint. The amount of dissolved gas was evaluated as the difference in the mass of the gas in the gas reservoir before the experiment and in the available gas phase after evaluation. The available gas phase in the measuring cell was determined as a difference of the total cell volume and the volume of the studied sample under equilibrium conditions (eqn (6)):

6 *m*_sol_ = *m*_res ini_ − *m*_res eq_ − *m*_cell eq_

where *m*_sol_ is the mass of the dissolved oxygen; *m*_res ini_ is the mass of oxygen in the thermostated gas reservoir before the experiment; *m*_res eq_ is the mass of oxygen in the thermostated gas reservoir under conditions of gas equilibrium with the studied liquid; *m*_cell eq_ is the mass of oxygen in the free volume of the measuring cell under equilibrium conditions with the studied liquid. The density of oxygen needed for the calculation of oxygen weight was computed with the help of RefProp model v. 10.0.^48^

The oxygen solubilities at 80 °C and 100 °C for co-solvent water mixtures were described using Sechenov eqn (7):^49^7
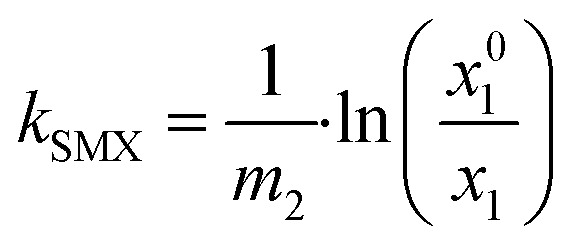
where *E*_2_ is the molality of the co-solvent in water, given in mol kg^−1^, *x*_1_^0^ is the mole fraction of oxygen in pure water, and *x*_1_ is the mole fraction of oxygen in the mixture of water and the co-solvent. The oxygen solubilities in pure water *x*_1_^0^ were measured up to 77 °C in this work. They were extrapolated to 100 °C and agree with the values reported by Crovetto^50^ within a standard deviation of 5%. Owing to this good agreement, the Sechenov parameters *k*_SMX_ were calculated using the values of *x*_1_^0^ and *x*_1_ obtained in this study.

### 5.4 Computational methods

The geometries of a model POM with either EtOH, *i*PrOH, *t*BuOH, MeCN or DMSO bound at 21 different positions (Fig. S39) were optimised using the r^2^SCAN-3c functional^51^ implemented in Orca 6.0.^52^ The solvation effects were accounted for using the conductor-like polarisable continuum model (C-PCM),^53^ using water as the solvent. The stationary points were confirmed as minima using harmonic vibrational frequency analysis. The binding free energies of the different co-solvents were calculated as the difference in the Gibbs free energies between the bound complexes (as described above) and a supermolecule geometry calculation, in which the HPA-2 and co-solvent were separated by at least 10 Å. Imaginary frequencies less than *iω* = 20 cm^−1^ were neglected for the supermolecule calculations only. Binding free energies of xylose to HPA-2 in the presence of (and absence of) the co-solvents above were calculated using the same approach.

## Conflicts of interest

There are no conflicts to declare.

## Data Availability

Additional data and x-y-z files for computational details can be found on 10.5281/zenodo.18267503.
